# Retained Fetal Bones: Rare cause of Abnormal Uterine Bleeding

**DOI:** 10.12669/pjms.36.ICON-Suppl.1719

**Published:** 2020-01

**Authors:** Farah Hameed, Samia Shuja

**Affiliations:** 1 Farah Hameed FCPS Department of Gynae & Obs, Sheikh Saeed Memorial Campus, The Indus Hospital Karachi, Pakistan; 2 Samia Shuja FCPS Department of Gynae & Obs, Sheikh Saeed Memorial Campus, The Indus Hospital Karachi, Pakistan

**Keywords:** Retained fetal bones, Abnormal uterine bleeding, Miscarriage, Dilatation and evacuation, Transvaginal sonography

## Abstract

Intrauterine retention of fetal bones is a rare complication of unsafe abortion. Patients may present with infertility, chronic pelvic pain, menorrhagia and vaginal discharge. Here, we discuss a management of young female patient with uterine bleeding after miscarriage. Other causes of abnormal uterine bleeding such as infection, polyp, fibroid, malignancy were excluded. Retained intrauterine fetal bones were found as a sole cause of her complaint. Diagnosis was made with pelvic ultrasound and pelvic radiography. Dilatation and curettage was performed to remove the fetal bones with a good subsequent outcome.

## INTRODUCTION

There are many causes of abnormal uterine bleeding (AUB) which include polyp, adenomyosis, leiomyoma, malignancy and hyperplasia, coagulopathy, ovulatory dysfunction, endometrial and iatrogenic causes as per FIGO (International Federation of Obstetrics and Gynaecology) classification.^1^ Intrauterine retention of fetal bone is a rare cause as the prevalence of retained fetal bones is approximately 0.15% among patients undergoing hysteroscopy.^2^ Common clinical presentations are infertility, chronic pelvic pain and vaginal discharge. Abnormal uterine bleeding is a rare presentation. Transvaginal sonography is an effective tool for diagnosis. Here we present a case of abnormal uterine bleeding secondary to retained fetal bone.

## CASE REPORT

A 25 years old lady P2+1, presented in the gynecology outpatient department with the complaint of abnormal uterine bleeding for the preceding one year. Her first two pregnancies were uneventful, both babies were alive and the last born was five years old. Her third pregnancy resulted in a missed abortion at 16 weeks followed by surgical evacuation of uterus at a private healthcare facility. She never experienced menstrual irregularity since menarche. After uterine evacuation, she was symptom free for a month or so. She then continued to have periods at regular intervals with variable intermenstrual bleeding. Bleeding used to be mild, painless and totally erratic. She never used any form of contraception. She went to different clinics for treatment. Her record showed that she had tried antibiotics, non steroidal anti inflammatory drugs (NSAIDs), tranexamic acid and progestogens but she could not get relief. She had a pelvic ultrasound done at a private facility which reported an intrauterine contraceptive device (IUCD) within the uterine cavity; despite there being no history of IUCD insertion.

On examination, her vital signs were within normal range, however she looked a little pale. Her thyroid was not enlarged. Abdominal examination was unremarkable. Speculum examination revealed healthy looking cervix and vagina. On bimanual examination uterus was bulky, anteverted, mobile and both lateral fornices were clear.

**Fig.1:**
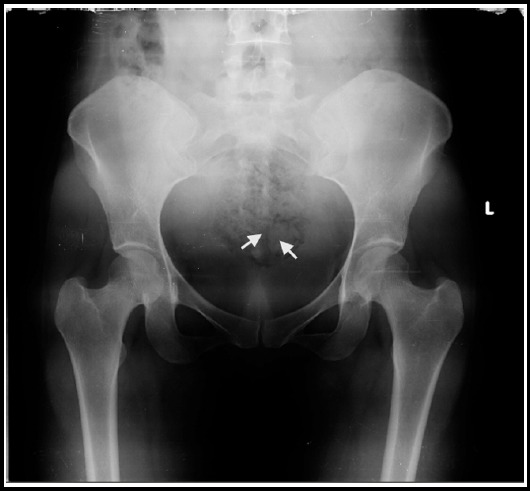
X-Ray pelvis showing radiopaque shadows.

Transvaginal sonography (TVS) was done by a trained sonologist. On TVS hyper-echogenic foci with suspicion of fetal bone were seen. The sonologist advised pelvic radiograph where radio opaque shadows confirmed her findings (Fig.1).

**Fig.2:**
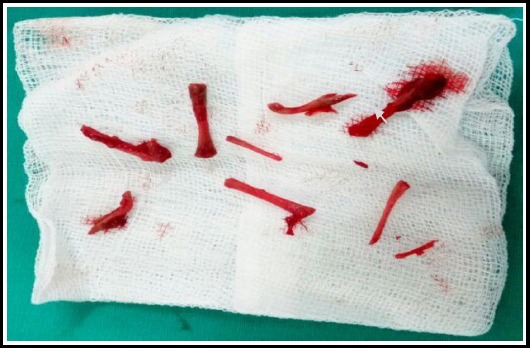
Fetal bones removed from uterine cavity.

As hysteroscopy is not available in our setting, so we planned dilatation and evacuation (D & E) under general anesthesia. The uterine cavity had a gritty feeling which was bony hard in places. Fourteen tiny fetal bones were removed from the uterine cavity with the help of sponge holding forcep and curette (Fig.2). Bleeding was minimal and the procedure was uneventful. The patient was discharged after 24 hours of observation.

She was followed in outpatient clinic for six months and her complaints settled. Fetal bones were found to be the sole cause of her abnormal uterine bleeding.

## DISCUSSION

Unsafe abortion is one of the most neglected healthcare problems in low middle income countries. It is estimated that 97% of about 20 million unsafe abortions occur in these regions. High incidence is mainly due to restrictive abortion laws coupled with low level of awareness and use of contraception.^3^

Retained fetal bone is a rare complication of unsafe abortion. Nearly 293 cases have been reported in literature including 16 cases from India.^4^ The incidence is 0.15% in patients undergoing diagnostic hysteroscopy.^2^

It is most common after evacuation of second trimester miscarriage. It should be considered in all patients with infertility, abnormal uterine bleeding, dysmenorrhea or vaginal discharge, dating from pregnancy termination.^5^ Diagnosis is often made during investigation of secondary infertility and accounts for 11.9 % of foreign body removed from uterine cavity.

Retention of fetal bone as a cause of abnormal uterine bleeding is rare.^6^ Lewis et al. measured menstrual blood volume and prostaglandin F2 concentration before and after removal of retained fetal bone. In women with infertility and menorrhagia, menstrual blood volume and total prostaglandin concentration decreased by 50% after removal of fetal bones.^7^ In our case abnormal uterine bleeding (AUB) probably occurred through the same mechanism.

Transvaginal sonography has remarkably improved the evaluation of pelvic pathology without recourse to invasive procedure. Any fetus that has attained at least 12 weeks of gestation is capable of endochondreal ossification. The bones appear hyperechogenic areas with posterior shadowing. This is important as deeply embedded bones are likely to be missed on hysteroscopy.^8^ Treatment of retained fetal bone is removal either through evacuation by conventional method or under hysteroscopic guidance. Relief of symptoms is dramatic.^9^

## CONCLUSION

In women presenting with abnormal uterine bleeding following a pregnancy termination, retained fetal bones should be considered as one of the causes. Although rare, a high index of suspicion is the only way to get to the correct diagnosis. Transvaginal ultrasound is an excellent tool for diagnosis. Radiographic examination of pelvis can have a supplemental role. Treatment is by removal of fetal bones. Patients become symptom free soon after treatment.

## References

[1] Munro MG, Critchley HO, Broder MS, Fraser IS, Disorders Fwgom (2011). FIGO Classification System (PALM-COEIN) For Causes Of Abnormal Uterine Bleeding In Nongravid Women Of Reproductive Age. Int J Gynaecol Obstet.

[2] Makris N, Stefanidis K, Loutradis D, Anastasiadou K, Hatjipappas G, Antsaklis A (2006). The Incidence Of Retained Fetal Bone Revealed In 2000 Diagnostic Hysteroscopies. J Soc Laparoendsc Surg.

[3] Bukar M, Geidam Ajfocgittbcfgul (2012). Complications Of Unsafe Abortion. J Found Clin Gynaecol Trop.

[4] Khan SN, Modi M, Hoyos LR, Imudia AN, Awonuga AO (2016). Bone In The Endometrium:A Review. Int J Fertil Steril.

[5] Chan NS (1996). Intrauterine Retention Of Fetal Bone. Aust N Z J Obstet Gynaecol.

[6] Kazakov BJ, Khankoev IM, Pererva VV (1994). Results Of Hysteroscopic Method Of Foreign Body Removal Out Of Uterus Cavity. J Am Assoc Gynecol Laparosc.

[7] Lewis V, Khan-Dawood F, King M, Beckmann C, Dawood MY (1990). Retention Of Intrauterine Fetal Bone Increases Menstrual Prostaglandins. Obstet Gynecol.

[8] Bakhshi PS, Allahbadia G, Kaur K, Virk SPS (2004). Hysteroscopic Removal Of Intrauterine Retained Fetal Bones. Gynecol Surg.

[9] Xiao S, Tian Q, Xue M (2014). Infertility Caused By Intrauterine Fetal Bone Retention:A Case Report. J Med Case Rep.

